# The PreS-Based Recombinant Vaccine VVX001 Induces Hepatitis B Virus Neutralizing Antibodies in a Low-Responder to HBsAg-Based HBV Vaccines

**DOI:** 10.3390/vaccines12101123

**Published:** 2024-09-30

**Authors:** Inna Tulaeva, Felix Lehmann, Nora Goldmann, Alexandra Dubovets, Daria Trifonova, Mikhail Tulaev, Carolin Cornelius, Milena Weber, Margarete Focke-Tejkl, Alexander Karaulov, Rainer Henning, David Niklas Springer, Ursula Wiedermann, Dieter Glebe, Rudolf Valenta

**Affiliations:** 1 Institute of Pathophysiology and Allergy Research, Center for Pathophysiology, Infectiology and Immunology, Medical University of Vienna, 1090 Vienna, Austria; inna.tulaeva@meduniwien.ac.at (I.T.);; 2 Laboratory of Immunopathology, Department of Clinical Immunology and Allergology, I.M. Sechenov First Moscow State Medical University, 119991 Moscow, Russia; 3 Institute of Medical Virology, National Reference Center for Hepatitis B Viruses and Hepatitis D Viruses, German Center for Infection Research (DZIF), Partner Site Giessen-Marburg Langen, Justus Liebig University, 35390 Giessen, Germany; felix.lehmann@charite.de (F.L.);; 4 Viravaxx AG, 1190 Vienna, Austria; 5 Center for Virology, Medical University of Vienna, 1090 Vienna, Austria; 6 Institute of Specific Prophylaxis and Tropical Medicine, Center for Pathophysiology, Infectiology and Immunology, Medical University of Vienna, 1090 Vienna, Austria; 7 NRC Institute of Immunology FMBA of Russia, 115552 Moscow, Russia; 8 Karl Landsteiner University of Health Sciences, 3500 Krems, Austria

**Keywords:** HBV vaccines, preS surface protein, HBsAg, non-responsiveness

## Abstract

*Background:* Approximately 10–20% of subjects vaccinated with HBsAg-based hepatitis B virus (HBV) vaccines are non-responders. BM32 is a recombinant grass pollen allergy vaccine containing the HBV-derived preS surface antigen as an immunological carrier protein. PreS includes the binding site of HBV to its receptor on hepatocytes. We investigated whether immunological non-responsiveness to HBV after repeated HBsAg-based vaccinations could be overcome by immunization with VVX001 (i.e., alum-adsorbed BM325, a component of BM32). *Methods:* A subject failing to develop protective HBV-specific immunity after HBsAg-based vaccination received five monthly injections of 20 µg VVX001. PreS-specific antibody responses were measured by enzyme-linked immunosorbent assay (ELISA) and micro-array technology. Serum reactivity to subviral particles of different HBV genotypes was determined by sandwich ELISA. PreS-specific T cell responses were monitored by carboxyfluorescein diacetate succinimidyl ester (CFSE) staining and subsequent flow cytometry. HBV neutralization was assessed using cultured HBV-infected HepG2 cells. *Results:* Vaccination with VVX001 induced a strong and sustained preS-specific antibody response composed mainly of the IgG_1_ subclass. PreS-specific IgG antibodies were primarily directed to the N-terminal part of preS containing the sodium taurocholate co-transporting polypeptide (NTCP) attachment site. IgG reactivity to subviral particles as well as to the N-terminal preS-derived peptides was comparable for HBV genotypes A–H. A pronounced reactivity of CD3^+^CD4^+^ lymphocytes specific for preS after the complete injection course remaining up to one year after the last injection was found. Maximal HBV neutralization (98.4%) in vitro was achieved 1 month after the last injection, which correlated with the maximal IgG reactivity to the N-terminal part of preS. *Conclusions:* Our data suggest that VVX001 may be used as a preventive vaccination against HBV even in non-responders to HBsAg-based HBV vaccines.

## 1. Introduction

Hepatitis B virus (HBV) causes acute and chronic liver infections, which often lead to long-term complications such as cirrhosis and liver cancer. Accordingly, HBV is one of the major public health threats. As reported by the WHO, there were 1.2 million new HBV infections in 2022, and the estimated number of deaths caused by HBV was around 1.1 million. Ongoing chronic HBV infection is a main driver for hepatocellular carcinoma, which represents 80% of all liver cancer cases, and is the third most common cause of cancer deaths [1,2]. Vaccination against HBV was introduced as the most important tool for the control of HBV morbidity around forty years ago, starting with the first generation of vaccines derived from plasma obtained from HBV-infected donors. Later, second-generation vaccines based on recombinant HBV surface antigen (HBsAg) produced in yeast became available and currently remain the basis for the internationally-accepted HBV immunization program, which has resulted in a significant decrease in the hepatitis B burden in a number of countries with high vaccine coverage. However, at least 10% of vaccinees fail to mount a protective anti-HBs response, which is defined as a level of anti-HBs antibodies ≥ 100 IU/L, ≥20 IU/L, or ≥10 IU/L, depending on different local regulations [3,4,5,6,7,8,9]. Anti-HBs antibody test systems can show some variability, which may need to be taken into account when deciding whether the boosting of immunity might be needed [10,11,12]. Among healthy individuals, the rate of non-responsiveness is estimated to be 5–15% in different studies [13], however, globally, it appears to be largely underestimated, since a significant proportion of the population has one or more low-response risk factors [14,15]. The latter include older age, obesity, smoking, and other chronic illnesses [16,17] as well as genetic predisposition determined by immune response genes in the major histocompatibility complex (MHC) [4,18,19,20,21,22,23]. The non-responsiveness to HBV vaccines is also influenced by the vaccine composition, adjuvant, and dosage [24,25]. Thus, it may be advisable that vaccinees who do not respond to the initial immunization course should be re-vaccinated, eventually with a different vaccine type [26,27,28]. However, since the currently available alternative vaccines are all based mainly on HBsAg, the chance of success is only low. Thus, other alternatives to the HBsAg-based standard (e.g., Engerix, Twinrix) or alternative (e.g., Fendrix, Heplisav-B) vaccines are needed, especially for individuals at high risk of HBV exposure.

Protection against HBV can be achieved by inducing antibodies against the viral envelope consisting of the three surface proteins encoded in one open reading frame (S-ORF) with different start codons: large hepatitis B surface protein (LHBs) composed of preS1, preS2 and S domains; middle hepatitis B surface protein (MHBs) composed of preS2 and S domains; and small hepatitis B surface protein (SHBs,) consisting only of the S domain. The term HBV surface antigen (HBsAg) is used to describe reactivity against the highly antigenic loop (AGL) of the S-domain (anti-HBs), especially for vaccines containing only SHBs (e.g., Engerix/Twinrix). While all three proteins serve as the fundamental components of the viral envelope, they also constitute the building blocks of subviral particles (SVP). These non-infectious particles are produced in surplus from HBV-infected cells and contain only the three surface proteins, with varying quantities. The surface protein domains preS (preS1 + preS2) and S are both involved in HBV entry to the liver cells, and are therefore reasonable vaccine candidates. Of those, the S domain, responsible for the initial low-specificity attachment to heparan sulfate proteoglycans, was discovered first and became the basis for conventional vaccination preventing the initial step of HBV entry [14]. It has been reported that preS2 binds to polymerized human albumin, and several studies have shown the virus neutralizing effect of anti-preS2 antibodies, however, it was later concluded that MHBs and a specific sequence of preS2 were not essential for HBV infectivity [14]. As discovered later, preS1, and in particular its myristoylated N-terminus, mediates the highly-specific binding to sodium taurocholate co-transporting polypeptides (NTCPs), a bile salt transporter on hepatocytes, and contains highly conserved amino acids essential for infection [29]. Thus, the preS1–NTCP interaction represents a potent target for HBV entry inhibition by blocking NTCPs with, for example, preS1-derived peptides or blocking the infectious viral particles with preS1-specific antibodies by passive or active immunization [30].

BM32 is a recombinant grass pollen allergy vaccine based on peptide-carrier technology that consists of the preS (preS1 + preS2, GenBank: AAT28735) protein as an immunological carrier fused to hypoallergenic peptides on both the N- and C-terminus. Designed initially for the allergen-specific immunotherapy of grass pollen allergy, BM32 has four compounds: BM321, BM322, BM325, and BM326, containing hypoallergenic peptides derived from the major Timothy grass pollen allergens Phl p 1, Phl p 2, Phl p 5, and Phl p 6, respectively [31]. Aside from hypoallergenic activity, good safety profile, the induction of allergen-specific blocking antibodies after basic immunization, which are further boostable by a single injection, and clinical efficacy for treating grass pollen-induced rhinitis have been shown in a series of clinical trials (ClinicalTrials.gov IDs: NCT01350635, NCT01445002, NCT01538979, NCT02643641), BM32 elicited preS-specific antibodies, which are directed mainly against the N-terminal part of preS1 and neutralized HBV in vitro, thus posing BM32 as an alternative HBV vaccine candidate, especially for non-responders to the standard HBsAg-based vaccination [32,33]. In this report, we show that the lack of anti-HBV protection can be overcome by immunization with VVX001, a vaccine containing alum-adsorbed BM325, in a subject with a long history of unsuccessful vaccinations with HBsAg-based vaccines.

## 2. Materials and Methods

### 2.1. Vaccine and Immunization Schedule

**Figure 1:**
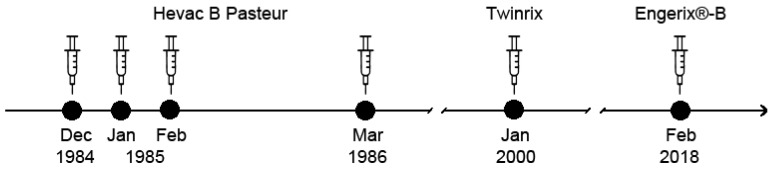
Vaccinations with conventional HBsAg-based vaccines in the study subject. Time points and administered vaccines are indicated.

**Figure 2:**
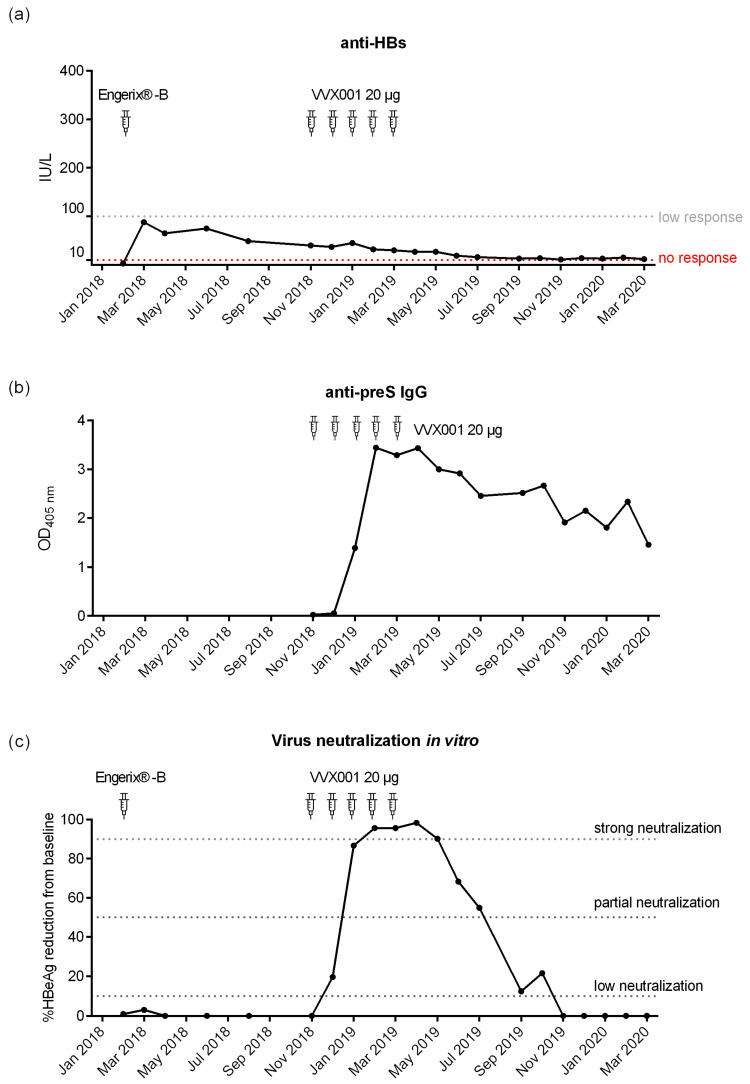
Development of antibodies to HBV surface proteins and their virus neutralization capacity. (**a**) Anti-HBs antibody levels (IU/L) measured after the last booster immunization with Engerix-B and after the preS-based vaccination. (**b**) PreS-specific IgG levels (OD) measured by ELISA after VVX001 immunization. (**c**) Percentages reduction in HBeAg secretion of infected NTCP-expressing HepG2 cells after pre-incubation of HBV inocula with sera obtained in the course of immunization compared to the baseline. Neutralization: ≥90% (strong neutralization), ≥50–90% (partial neutralization), ≥10–50% (low neutralization).

The subject of the present report (male, born on 25 February 1963, medical doctor, and researcher) has been vaccinated with licensed HBsAg-based vaccines six times, as described in Figure 1. The basic immunization was performed with the plasma-derived vaccine Hevac B Pasteur [34]. Subsequent booster injections were the combined hepatitis A and B vaccine Twinrix (20 µg HBsAg, yeast-derived, GlaxoSmithKline Biologicals, Rixensart, Belgium) and Engerix-B vaccine (20 µg HBsAg, yeast-derived, GlaxoSmithKline). After the last immunization with Engerix-B on 8 February 2018, anti-HBs levels were monitored at 1, 2, 4, 6, and 9 months after vaccination followed by self-immunization with VVX001 (Viravaxx, Vienna, Austria), as shown in Figure 2a.

BM325, a component of the BM32 vaccine, is a recombinant fusion protein composed of the preS (HBV genotype A2, GenBank: AAT28735) of LHBs and peptides derived from the grass pollen allergen Phl p 5. BM325 was produced by Biomay AG (Vienna, Austria) according to GMP standards, and aluminum hydroxide was used as the adjuvant [31]. The vaccine VVX001 containing alum-adsorbed BM325 was applied by the subject himself subcutaneously (20 µg per injection) in the non-dominant upper arm (deltoid muscle region) at each of the following time points: 19 November 2018, 14 December 2018, 11 January 2019, 7 February 2019, and 8 March 2019. The dose of 20 µg and the number of injections were determined based on the data from the previous BM32 clinical trials (ClinicalTrials.gov IDs: NCT01350635, NCT01445002, NCT01538979, NCT02643641). Blood samples were obtained at the time points depicted in Figure 2a.

### 2.2. Serological Assays

The anti-HBs and anti-HBc antibody concentrations as well as the levels of HBsAg and HBeAg were determined using commercial enzyme-linked immunosorbent assays (ELISAs, Dia.Pro Diagnostics, Sesto San Giovanni, Italy). Anti-HBs antibodies were quantified in international units per liter (IU/L). Additionally, HBV-DNA was determined in sera by polymerase chain reaction (PCR), using the commercial Aptima^®^ HBV Quant Assay (Hologic Inc., San Diego, CA, USA) [35] and quantified in international units per milliliter (IU/mL). All tests were performed following the manufacturers’ instructions and their features are listed in Table S1.

Recombinant preS (preS1 + preS2) and preS-derived synthetic peptides spanning the whole preS sequence of HBV genotype A2 (GenBank: AAT28735) as well as peptides covering the NTCP attachment site of genotypes A–H (Table S2) were produced as described [33]. The preS- and peptide-specific IgG, IgG_1–4_, IgM, IgA determination as well as IgG_1_ and IgG_4_ quantification were performed by ELISA as described in [33]. If not stated otherwise, plots and charts were created using Prism 6.0 software (GraphPad, San Diego, CA, USA).

### 2.3. PreS Micro-Array Production and Sample Analysis

Glass slides containing six micro-arrays formed by oval epoxy frames (Paul Marienfeld GmbH & Co. KG, Lauda-Königshofen, Germany) were coated with amino-reacting polymer MCP-2 (Lucidant Polymers, Sunnyvale, CA, USA). The recombinant preS and preS-derived synthetic peptides were diluted and re-buffered for the following spotting conditions: concentration 0.5–1 mg/mL, buffer 75 mM Na_2_HPO_4_, pH 8.4. Afterward, they were spotted in triplicate on the pre-activated glass slides with a SciFlexArrayer S12 (Scienion AG, Berlin, Germany), as described in [36]. The micro-array layout is depicted in Figure S1.

For specific antibody detection, micro-arrays were washed for 1 min with phosphate-buffered saline containing 1% Tween 20 (PBST) and dried with a Sigma centrifuge and MTP-11113 rotor (both Sigma Laborzentrifugen GmbH, Osterode am Harz, Germany). For IgG, IgG_1_, and IgG_4_, IgM detection serum was diluted 1:100 and for IgA detection, 1:20 with a sample diluent (ImmunoCAP Specific IgA/IgG Sample Diluent, Phadia Ab, Uppsala, Sweden). Aliquots of 30 µL were added per array and incubated for 2 h at 22 °C. After another washing step, corresponding detection antibodies labeled with DyLight 550 (Cat. 62269, Pierce, IL, USA) with a concentration of 1.8 µg/mL targeting various types of immunoglobulins were applied: IgG (Cat. 009-000-003, Jackson ImmunoResearch, West Grove, PA, USA), IgG_1_ (Cat. 555868, BD Biosciences, Franklin Lakes, NJ, USA), IgG_4_ (Cat. 555878, BD), IgA_1_/A_2_ (Cat. 555886, BD), IgM (Cat. 555856, BD), and incubated for 30 min at 22°C. After incubation, the micro-arrays were washed with PBST and ddH_2_O to remove unbound antibodies and salts and then scanned with a Tecan Powerscanner (Tecan Trading AG, Männedorf, Switzerland). Detection was performed as described in [36]. MAPIX version 8.5.0 (Innopsys, Carbonne, France) software was used to analyze the scanned images. The specific antibody levels are expressed in fluorescence intensity (FI) units, and the FI of the sample diluent alone was subtracted for each antigen. Depicted are the means of triplicate measurements.

### 2.4. Determination of Antibody Reactivity to HBV Subviral Particles

In order to produce subviral particles (SVPs) consisting of HBV envelope proteins, HuH7 cells were transfected with sub-genomic plasmids encoding the HBsAg (LHBs, MHBs, SHBs) of isolates of genotypes A2 (N4879; WHO panel No. 3), B2 (N4214; WHO panel No. 4), C2 (N3825; WHO panel No. 9), D1 (N4203; WHO panel No. 10), E (N4881; WHO panel No. 13) described in Chudy et al., 2012 [37], F4 (GenBank: KY382411), H (V1688; WHO panel No. 15/H) described in Chudy et al., 2011 [38] as well as D3 (SHBs only, GenBank: NC_003977) under endogenous viral promoters. The cell culture supernatant was collected from day 2 to day 6 post transfection and the cell-cleared supernatant was concentrated by a factor of 50 (Vivaspin 20, 100,000 MWCO, Sartorius, Göttingen, Germany). The total HBsAg content of concentrates was determined with the quantitative Architect HBsAg assay (Abbott Laboratories, Wiesbaden, Germany).

Antibody reactivity to SVPs was determined by sandwich ELISA in the subject’s pre-immune serum, in a serum sample obtained on 5 April 2019 and in serum samples from two control subjects who had been vaccinated with HBsAg-based vaccines (subject 1: anti-HBs > 4000 IU/L); subject 2: anti-HBs > 6500 IU/L). Ninety six-well microplates (Cat. 675061, Greiner Bio-One, Kremsmünster, Austria) were coated with 0.2 µg/well HB1 mAb [39] in bicarbonate buffer (pH 9.6) overnight at 4 °C, washed twice with 100 µL/well PBS 0.05% Tween 20 (PBST), and residual binding sites were blocked with 100 µL/well 3% BSA/PBST for 5 h at 37 °C. After washing the plates twice, they were incubated with 0.1 µg/well SVPs in the HuH7 cell culture supernatant diluted with 0.5% BSA/PBST overnight at 4 °C, washed three times, and incubated with 50 µL/well serum diluted 1:10 in 0.5% BSA/PBST overnight at 4 °C. After washing three times with PBST, detection was performed by the addition of 50 µL/well horseradish peroxidase (HRP)-labeled anti-human IgG (Cat. 555788, BD Biosciences, Franklin Lakes, NJ, USA) diluted 1:3000 in 0.5% BSA/PBST and incubated on the plate for 2 h at 37 °C. The plates were then washed three times and the reaction was developed by incubation with the substrate solution: 1 mg/mL 2,2′-Azino-bis(3-ethylbenzothiazoline-6-sulfonic acid) di-ammonium salt (ABTS) (Cat. A1888, Sigma-Aldrich, Burlington, MA, USA) in 70 mM citrate-phosphate buffer containing 0.003% H_2_O_2_ (Cat. H1009, Sigma-Aldrich); absorbance was measured at a wavelength of 405 nm (reference wavelength 492 nm) on a Tecan Infinite F50 spectrophotometer (Tecan Trading AG, Männedorf, Switzerland Switzerland). ELISA was controlled by the omission of either part of the sandwich and buffer control, and the background reactivity of the HB1 antibody with each of the serum samples was subtracted. The values are displayed as the means of duplicate measurements of the optical density (OD) values with deviations of less than 5%.

### 2.5. Virus Neutralization Assay

A brief summary of the in vitro virus neutralization assay is depicted in Figure S2. In order to generate the infectious HBV preparation in vitro, a human hepatoma cell line (HepG2) was stably transfected with a plasmid encoding a 1.1 over-length HBV genome of genotype D3 (GenBank: NC_003977). Cells were incubated at 37 °C, 5% CO_2_ in William’s E medium (Thermo Fisher Scientific, Schwerte, Germany) containing 2% dimethyl sulfoxide (DMSO; Carl Roth, Germany), 2% fetal calf serum (FCS; Thermo Fisher Scientific), 1 µg/mL doxycycline (Carl Roth), 1 µg/mL G418 (Carl Roth), and 1 µg/mL puromycin (Carl Roth) for several weeks with continuous media exchanges. The supernatant was collected and ultra-filtrated to enrich viral particles. The viral load was determined to be 3.7 × 10^11^ Ge/mL by a highly-sensitive qPCR, as previously described [40]. HBsAg in the viral stock was determined to be 4080 IU/mL by the quantitative Architect HBsAg assay (Abbott Laboratories, Wiesbaden, Germany).

Next, HepG2-NTCP cells [41] were pre-treated with 6 µg/mL doxycycline to induce NTCP expression at least 3 days prior to infection and subsequently seeded into 24-well plates the day before infection. Cells were infected with virus inoculum (5 × 10^9^ Ge; 55.9 IU HBsAg) in hepatocyte growth medium (HGM) consisting of William’s E medium supplemented with 1× insulin-transferrin-selenium (Thermo Fisher Scientific, Germany), 2  mM L-glutamine (Thermo Fisher Scientific), 100  µg/mL gentamicin (Thermo Fisher Scientific), 10  nM dexamethasone (Sigma-Aldrich, Germany), 1  mM sodium pyruvate (Thermo Fisher Scientific), 0.2% bovine serum albumin (BSA; Carl Roth, Karlsruhe, Germany) in the presence of 4% PEG-8000 (Sigma-Aldrich), 2% DMSO (Carl Roth), and 100 ng/mL EGF (PeproTech, Hamburg, Germany) as a final concentration (f.c.) each. NTCP expression was routinely controlled by uptake of the green-fluorescent bile salt NBD-TC (4-nitrobenzo-2-oxa-1,3-diazole taurocholic acid), as previously described [42].

For neutralization, virus inoculum was pre-incubated with sera at defined concentrations in a dilution with HGM for 1 h at 37 °C and under regular agitation. Pre-incubation with mouse-derived monoclonal anti-preS1 antibody MA18/7 detecting an epitope DPXF in the preS1 amino acids 20 to 23 (31 to 34 in genotype A) [43] was performed in the same manner and served as the neutralization control. Twenty hours after virus inoculation, cells were washed three times with HGM and further incubated for 9 days in HGM supplemented with 2% DMSO with the medium changed every 2 days. As a first outcome parameter of the HBV virus neutralization assay, HBeAg secreted from infected cells into the cell culture supernatant was determined in the supernatants collected from days 7 to 10 post infection (p.i.) using the Architect HBeAg assay (Abbott Laboratories, Wiesbaden, Germany).

As a second outcome parameter, the immunofluorescence of the infected cells was measured as follows. At day 10 p.i., cells were fixed by incubation with 3.7% formaldehyde in PBS for one hour at 4 °C, washed with PBS, and permeabilized using 0.2% Triton X-100 in PBS for 30 min at room temperature (RT). Cells were washed again and blocked with 10% FCS in PBS for 60 min at RT. As the primary antibody, a polyclonal α-HBc antiserum from an immunized guinea pig (Eurogentec, Seraing, Belgium) was used in a 1:500 dilution for 1 h at 37 °C and the cells were then washed five times. For detection, cells were incubated with Alexa-488 coupled α-guinea-pig-IgG-antibodies (Thermo Fisher Scientific, Germany) in a 1:400 dilution for 30 min at 37 °C in the dark and subsequently washed five times. Nuclei were stained by the incubation of cells with 4′,6-diamidino-2-phenylindole (DAPI, 1 µg/mL f.c.) for one hour at 37 °C in the dark. The analysis of immunofluorescence was performed with the ImageXpress Pico automated cell-imaging system (Molecular Devices, San Jose, CA, USA). The total area analyzed was 12% of the total well size.

IC_50_ values were determined using the integrated “Absolute IC_50_, X is concentration” analysis integrated into GraphPad Prism version 9.4.1. Results are displayed as IC_50_ (±95% CI). In the neutralization experiments, the serum from a subject immunized with HBsAg-based vaccines containing 2600 IU/L anti-HBs was included for control purposes.

### 2.6. CD4^+^/CD8^+^ T Cell Proliferation Assay and Cytokine Responses

Peripheral blood mononuclear cells (PBMCs) were freshly isolated from heparinized blood by the Ficoll-Paque™ Plus density gradient (Cat. 17-1440-03, GE Healthcare, Chicago, IL, USA) and stained with a carboxyfluorescein diacetate succinimidyl ester (CFSE) (Cat. C1157, Invitrogen, Waltham, MA, USA) as described [44]. Aliquots of 10^5^ PBMCs in 200 µL of UltraCulture™ Serum-free Medium (Cat. BE12-725F, Lonza, Basel, Switzerland) supplemented with 2 mM L-glutamine (Gibco, Grand Island, CA, USA), 50 µM β-mercaptoethanol (Gibco), and 0.1 mg gentamicin (Gibco) per 500 mL were incubated for 7 days at 37 °C in a humidified atmosphere with 5% CO_2_ in Nuncclon 96-well plates (Cat. 163320, Thermo Fisher Scientific, MA, USA) in triplicate in the presence of 0.15 µg/well of recombinant preS, preS-derived peptides, or a mix of peptides P1–P8 equimolar to preS. Aliquots of 0.5 μL/well of Dynabeads Human T-Activator anti-CD3/CD28 (Cat. 11161D, Thermo Fisher Scientific) were used as positive controls; the medium alone served as a negative control. On day 8, flow cytometry analysis and gating were performed as described [32]. The levels of IL-2, IFN-γ, IL-4, IL-5, IL-10, IL-13, GM-CSF, and TNF-α were measured in the supernatants of seven-day PBMC cultures by the Luminex multiplex assay (Bio-Plex ProTM Human Cytokine 9-Plex Assay, Bio-Rad, Hercules, CA, USA) according to the manufacturer’s instructions using the Bio-Plex^®^ 200 System (Bio-Rad). For analysis, the median of the medium-only wells was subtracted from each of the triplicates cultivated with antigen, and the values displayed are the means of the triplicates.

## 3. Results

### 3.1. Repeated Vaccination with HBsAg-Based Vaccines Induced Only Low Anti-HBs Responses in the Study Subject

After basic immunization with the HBsAg-based vaccine Hevac B Pasteur and subsequent booster injections with Twinrix and Engerix-B (Figure 1), no robust anti-HBs antibody response could be established in the study subject. The subject was 23 years old at the time of completion of the first four-dose immunization schedule and completely healthy. The subject was also not affected by any medical condition or medication intake during the whole further observation period (i.e., until March 2020). The subsequent booster vaccinations with licensed HBsAg-based vaccines Twinrix (January 2000) and Engerix-B (February 2018) elicited only low levels of HBs-specific antibodies (anti-HBs; i.e., <100 IU/L after Engerix B), waning quickly during 2018 as depicted in Figure 2a and showing no virus neutralization before VVX001 immunization (Figure 2c).

### 3.2. VVX001 Induces Robust HBV-Specific Antibody Responses Directed Mainly to the N-Terminus of the preS-Containing NTCP Binding Site

Subcutaneous immunization with VVX001 was started in November 2018. No relevant immediate or late-phase side effects were observed after each of the five injections containing 20 µg VVX001 adsorbed to aluminum hydroxide when applied in monthly intervals. The vaccination with VVX001 induced a strong and sustained preS-specific IgG response that already peaked after the third immunization (Figure 2b and Figure S3) as well as distinct increases in Phl p 5-specific IgG antibodies (Figure S4). In order to exclude that the antibody increase was caused by clinically silent HBV infection, we performed HBV screening of the samples before and after VVX001 immunization; no signs of a possible HBV infection were observed (Table S2).

**Figure 3:**
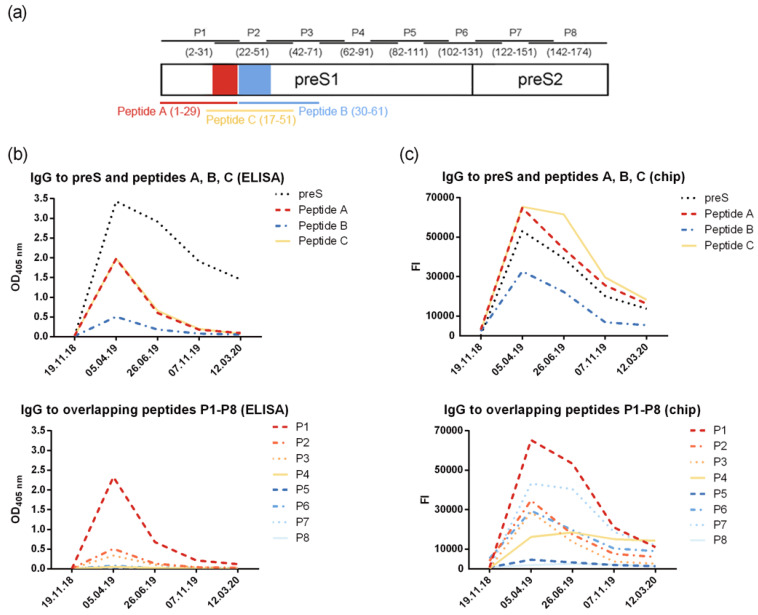
VVX001-induced IgG antibodies react mainly with the N-terminus of preS1. (**a**) Localization scheme of preS-derived peptide within the preS sequence (amino acid numbering indicated for genotype A) and the levels of preS/peptide-specific IgG measured by (**b**) ELISA (OD) and (**c**) micro-array technology (fluorescence intensity, FI).

The analysis of preS-specific antibody isotypes and subclasses showed that the preS-specific antibody response was composed mainly of IgG_1_ (Figure S3). The epitope specificity of the induced antibodies assessed with overlapping synthetic peptides P1–P8 (Figure 3a) showed that IgG antibodies were mainly directed to the N-terminal part of preS that contains the liver cell attachment site of HBV (Figure 3b). Antibodies were measured by ELISA and by micro-array technology.

The design of the preS micro-array containing preS and preS-derived peptides (six micro-arrays on each glass slide) and images of the scanned images are depicted in Figure S1. The results from the micro-array measurements were in good agreement with the results obtained by ELISA and showed a similar pattern of IgG reactivity focusing mainly on the N-terminus of preS. However, stronger signals were obtained with the micro-array for the peptides compared to the complete preS protein (Figure 3b,c). The levels of preS-specific IgM and IgA (Figure S5) were low and also directed mainly toward the N-terminal peptides of preS.

The micro-array measurements of preS- and peptide-specific IgG_1_ and IgG_4_ confirmed that IgG_1_ was the dominating IgG subclass with maximal levels present 1 month after the last injection of VVX001 (Figure S6a). The induction of IgG_4_ also became visible, but followed a different kinetic. The build-up phase of IgG_4_ was longer, and the maximal antibody levels were observed 3 months later than for IgG_1_ (i.e., 4 months after the last VVX001 injection) (Figure S6b). The quantification of IgG_1_ and IgG_4_ specific to preS showed that the preS-specific IgG_1_ serum concentration reached 1.9 mg/mL one month after the last injection and preS-specific IgG_1_ at a concentration of 0.68 mg/mL was still present one year after the course of immunizations (Table 1). The concentrations of preS-specific IgG_4_ were approximately 100-fold lower and, accordingly, could not be detected after the single immunization (Table 1). Nonetheless, preS-specific IgG_4_ were still detectable by micro-array measurements one year later (Figure S6b). The quantification of the N-terminal peptide-specific antibody levels suggested that the induced antibodies reacted to both the N-terminal epitopes of preS, which is important for HBV infectivity, with most of the reactivity directed toward the region defined by peptide A (Table 1).

### 3.3. VVX001-Induced Antibodies Cross-React with HBV Genotypes A-H

**Figure 4:**
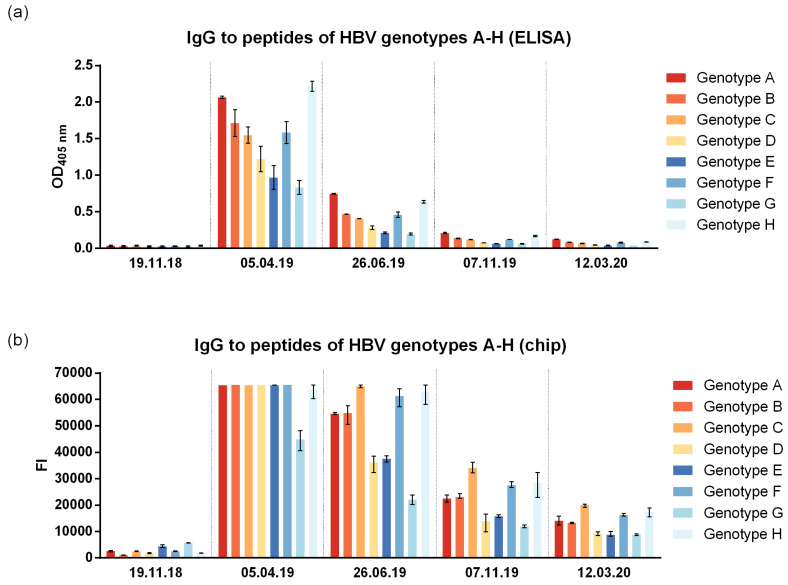
VVX001-induced antibodies cross-react to the NTCP binding site of preS of HBV genotypes A–H. Shown are the IgG levels to the synthetic peptides representing the NTCP attachment site of HBV genotypes A–H measured by (**a**) ELISA (OD) and (**b**) micro-array technology (fluorescence intensity, FI).

**Figure 5:**
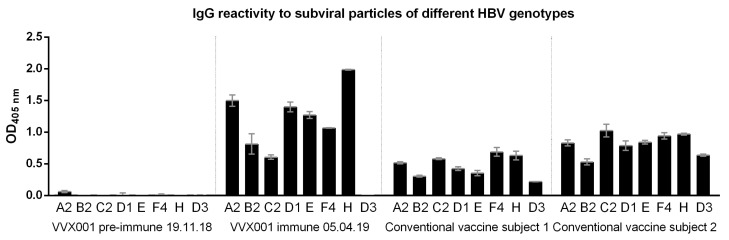
VVX001-induced antibodies react to SVPs of different HBV genotypes. Shown are IgG levels (OD) to SVPs of HBV genotypes A2, B2, C2, D1, E, F4, H (LHBs, MHBs, SHBs), and D3 (SHBs only) in the serum samples of the study subject before and after immunization as well as in two subjects successfully vaccinated with conventional HBsAg-based vaccines.

To address the question of genotype cross-reactivity, we synthesized eight peptides representing the liver cell attachment site of HBV genotypes A–H (Table S1) as described [33]. According to both the ELISA and micro-array measurements, IgG cross-reactivity to the eight peptides representing the NTCP attachment site was at all time points detectable for genotypes A–H, with somewhat lower reactivity to genotypes D, E, and G (Figure 4). The genotype cross-reactivity was also assessed using recombinant SVPs composed of large, middle, and small HBV surface proteins (LHBs, MHBs, SHBs) of HBV genotypes A2, B2, C2, D1, E, F4, and H as well as control SVPs composed of genotype D3 SHBs only (Figure 5). The pre-immune serum of the subject of the current study did not show any SVP reactivity before VVX001 immunization. The sample obtained one month after the last immunization reacted to all SVPs tested with the exception of the control SVPs of genotype D3 that contained no preS. Both of the two conventionally vaccinated subjects who had received the standard HBsAg-based immunization and had high anti-HBs levels within two years prior to the blood donation (subject 1: anti-HBs > 4000 IU/L, subject 2: anti-HBs > 6500 IU/L) reacted comparably to all SVPs including control genotype D3. Serum obtained from the study subject obtained one month (5 April 2019) after the last immunization with VVX001 showed higher reactivity to all LHBs-containing SVPs than the sera from the two control subjects (Figure 5).

### 3.4. VVX001-Induced Antibodies Strongly Neutralize HBV Infection In Vitro

A neutralizing ability of the antibodies induced by the vaccination was assessed for the samples obtained from the subject after vaccination with Engerix (February 2018) as well as before, during, and after immunization with VVX001 (Figure 2c). A well-established diagnostic HBeAg assay was used to determine in vitro virus neutralization with a high range between positive controls (i.e., HBV infection without the addition of serum “PC” HBeAg: 158.82–166.09 S/CO (signal/cutoff)) and the negative control (i.e., uninfected cells HBeAg < 1 S/CO). There was no relevant increase in virus neutralization after the last HBsAg booster injection with Engerix-B from February 2018 to November 2018 (Figure 2c). Immunization with VVX001 resulted in a strong increase in HBV neutralization with a strong neutralization observed between the time points February 2019 (1 month after the 3rd VVX001 injection) and May 2019 (2 months after the 5th VVX001 injection). The subsequent samples showed a decrease in neutralization potential but neutralization effects were observed until October 2019. After October 2019, no virus neutralization was observed in the in vitro virus neutralization test, although anti-preS antibodies were still present (Figure 2b,c).

**Figure 6:**
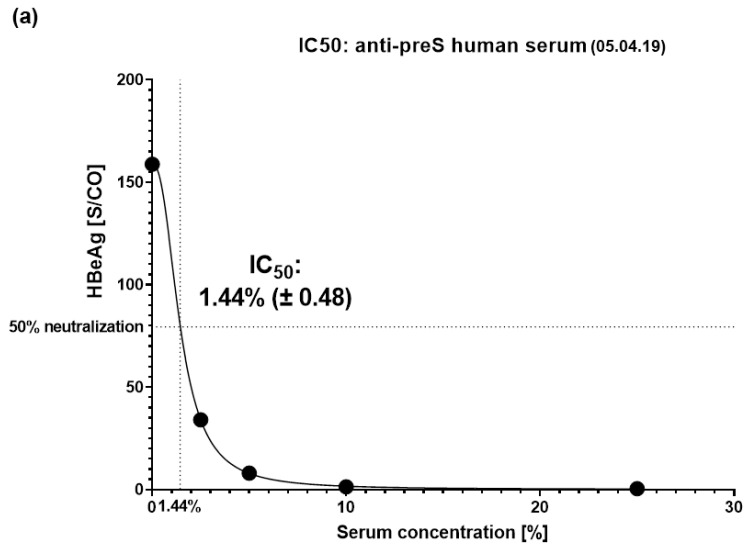

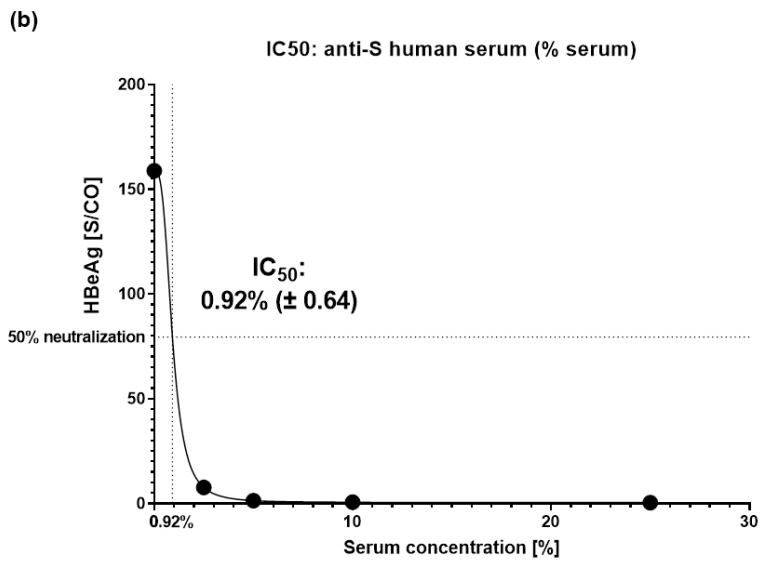
IC_50_ determination. IC_50_ values for serum dilutions determined based on the HBeAg results are presented with a 95% confidence interval for (**a**) the anti-preS-positive human serum obtained in April 2019 and (**b**) for the anti-S-positive human serum (conventional vaccine, 2600 IU/L anti-HBs).

Next, titration experiments were performed to determine the IC_50_ values (Figure S7). For titration, the serum obtained 1 month after the last VVX001 injection on 5 April 2019 was used and showed an IC_50_ corresponding to 1.44% (±0.48) of serum in the virus pre-incubation mix (Figure 6a). As a control, an immune serum containing 2600 IU/L of anti-HBs antibodies after conventional vaccination was used, which yielded an IC_50_ corresponding to 0.92% (±0.64) of serum in the virus pre-incubation mix (Figure 6b). Based on this comparison, we estimated that the subject’s serum taken in April 2019 contained neutralizing antibodies “functionally” corresponding to approximately 1661 IU/L of anti-HBs antibodies. The monoclonal anti-preS1 antibody MA18/7 was already 97% neutralizing at 1 µg/mL and fully neutralizing at all higher concentrations (Figure S7a,b). Therefore, the calculation of the IC_50_ of MA18/7 could not be performed.

In fact, an even higher virus neutralization was measured in the subject of this study after another course of preS-based self-vaccination with a preS-RBD SARS-CoV-2 vaccine as described [45]. After immunization with the preS-RBD vaccine, a 98.6% HBV neutralization (HBeAg 1.185 S/CO in the tested sample vs. HBeAg 83.63 S/CO at baseline) was observed, indicating that it is possible to boost the preS-specific virus-neutralizing antibody response through repeated immunizations.

### 3.5. VVX001 Induces a Sustained preS-Specific CD3^+^CD4^+^ Cellular Response Which Is Accompanied by a Mixed Th2/Th1 Cytokine Response

**Figure 7:**
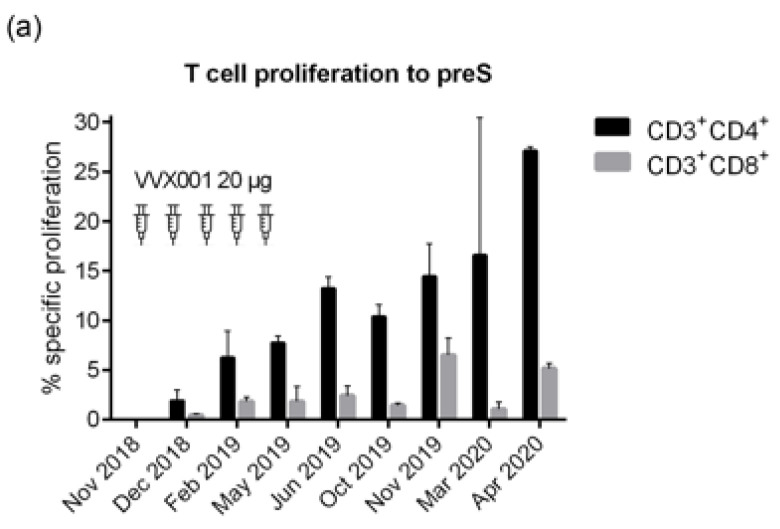

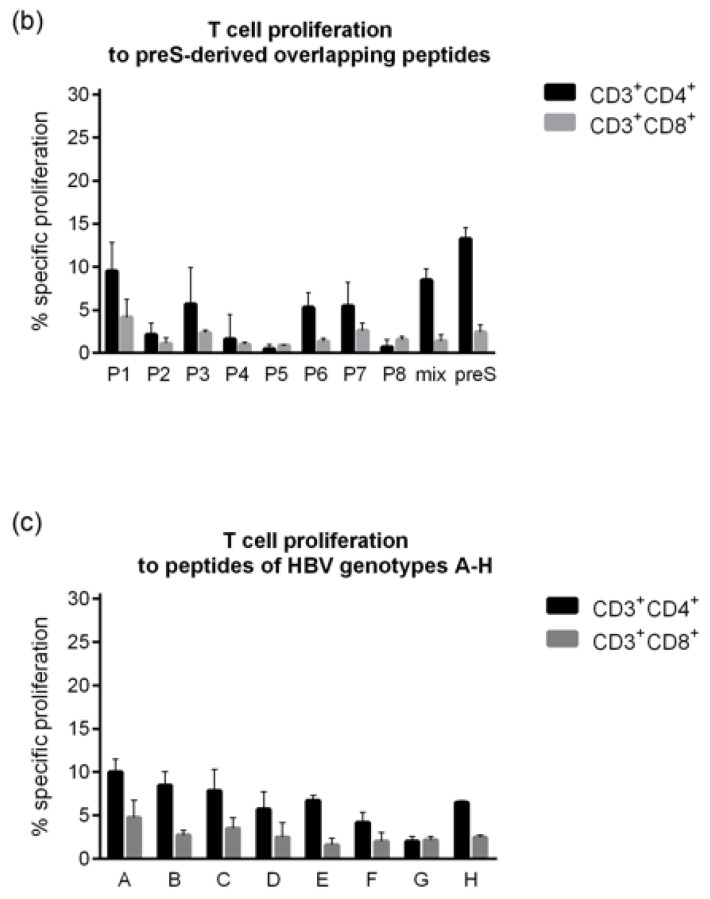
PreS-specific T cell responses after vaccination with VVX001. Shown are the percentages of proliferated CD3^+^CD4^+^ and CD3^+^CD8^+^ cells upon stimulation with (**a**) preS over time, (**b**) preS, P1-P8, equimolar mix of P1–P8, and (**c**) peptides representing the NTCP attachment site of genotypes A–H at the time point 4 months after the last injection (26 June 2019).

Vaccination with VVX001 induced a growing and sustained preS-specific CD4^+^ T cell and to a much lower degree of a preS-specific CD8^+^ T cell response in the study subject (Figure 7a). Interestingly, peptide 1 derived from the N-terminus of preS, which was identified as a major B cell epitope, was also identified as a T cell epitope-containing peptide (Figure 7b). All peptides derived from the NTCP attachment site of HBV genotypes A–H induced the proliferation of CD4^+^ > CD8^+^ T cells from the vaccinated subject, although the proliferation stimulated with peptides from genotypes D, F, G, and H was low (Figure 7c).

Figure S8 shows the induction of preS-specific cytokine responses induced in cultured PBMCs obtained before and after immunization with VVX001 in the study subject. We found that immunization with VVX001 induced a mixed preS-specific Th2/Th1 cytokine secretion profile, which was characterized by the induction of IL-4, IL-5, IL-13, and GM-CSF, on the one hand, and by the induction of IFN-γ on the other hand (Figure S8). We also noticed the development of preS-specific IL-2 and TNF-α responses and the development of an eventually tolerogenic IL-10 response (Figure S8).

## 4. Discussion

Previously, we developed a platform for recombinant allergen-specific immunotherapy vaccines based on recombinant fusion proteins containing HBV-derived preS as immunological carrier protein fused to hypoallergenic allergen derived peptides [46]. PreS was selected as the carrier protein for these allergy vaccines because it contains the binding site of HBV to its cognate receptor NTCP on liver cells, and hence was expected to generate antibodies upon immunization that may also protect against HBV infections. Indeed, we found that patients who were allergic to grass pollen and had been immunized with BM32, a grass pollen allergy vaccine containing four preS-fusion proteins, BM321, BM322, BM325, and BM326, developed preS-specific antibodies, which were able to neutralize HBV infections in vitro [32,33]. Accordingly, BM32 and its components were considered as vaccine candidates against HBV infections. To the best of our knowledge, BM32 is the first and still the only recombinant vaccine that has been used for the active immunization of humans and is based solely on preS [31,47]. A number of vaccine candidates contain preS1 and/or preS2, mainly in combination with HBsAg or other HBV antigens, but very few have advanced to market, among which are the so-called third-generation vaccines PreHevbrio (also known as Sci-B-Vac, Bio-Hep-B, Hepimmune) and Hepacare (other name Hepagene, discontinued), both produced in mammalian cells containing a low portion of preS when compared to its content of HBsAg [48,49,50].

The aim of the present self-experiment was to induce a protective immune response against HBV in a medical doctor and researcher with regular contact to blood products (R.V.). The study subject had been vaccinated six times with HBsAg-based vaccines without achieving protective HBV-specific immunity, as documented by the lack of formation of HBV-neutralizing antibodies. Even after the last HBsAg-based vaccination, only a very low and rapidly declining HBV-specific antibody response not exceeding 60 IU/L was achieved. Thus, the achieved seroconversion at a low level (anti-HBs < 100 IU/L) is considered as unsuccessful vaccination according to the current guidelines [15,48,51].

Since conventional HBsAg-based vaccines did not seem to ensure protection against HBV, self-immunization with the preS-based recombinant vaccine VVX001 was performed by the volunteer in accordance with the Declaration of Helsinki. The vaccine was well-tolerated. The maximal anti-preS level as well as strong increase in HBV neutralization was already observed after the third injection (Figure 2), which suggests that a short basic immunization might be sufficient. The increase in virus neutralization was associated with the induction of IgG antibodies to the N-terminal part of preS (represented by peptides A, B, C, P1, P2, P3, and peptides of genotypes A–H) (Figure 3, Table 1) and to the whole preS protein, which were still strongly detectable 1 year after the injection course in the absence of virus neutralizing ability in the serum. Generally, mostly N-terminal preS1 peptides, but also preS2 N-terminus peptides (P6, P7), were recognized by both antibodies and T cells, corresponding to the regions with described HBV neutralizing epitopes [14,52].

We have already previously reported the results from proof-of-principle work demonstrating the HBV neutralization capacity of BM32-induced antibodies in a random population of subjects allergic to grass pollen [32] as well as a detailed report on its epitope mapping, cross-reactivity to NTCP binding site of genotypes A–H, and quantification of serum antibody concentrations [33,53]. This study confirms our previous observations but also contains several new important findings.

First, we demonstrated that VVX001 can induce an HBV-neutralizing antibody response in a low-responder to classical HBV vaccines. Second, we showed that the HBV-neutralizing antibody response could be obtained with a relatively low dose of BM antigen, while the doses administered in the previous allergy trials included two, four, and eight times more preS than in our current study. Third, the HBV genotype cross-reactivity was assessed not only with synthetic linear peptides, but also with SVPs containing all three HBV surface proteins in a three-dimensional conformation analogous to that of infectious virions. Furthermore, we reported the development of a micro-array containing preS and preS-derived peptides that turned out to be useful for analyzing preS-specific antibody responses with small sample volumes (i.e., approx., 1 μL per test/array).

Immunization with VVX001 induced a robust anti-preS IgG response that mainly consisted of a preS-specific IgG_1_ subclass, but preS-specific IgG_4_ development was also observed. As for many other vaccines that are administered, the specific antibody levels declined within a year after immunization [54,55,56,57,58]. However, it is quite likely that the preS-specific IgG_4_ response would be boosted by further immunizations to achieve a sustained specific IgG_4_ response, as was observed in earlier allergen-specific immunotherapy trials with BM32 [33,54]. Moreover, the assessment of T cell proliferation revealed the protracted impact on the cellular response, suggesting that VVX001 vaccination has generated sufficient cellular memory to provide the basis for long-term protective immunity [59,60].

A comparison of the efficacy of the VVX001-induced preS-specific antibodies with successful conventional vaccination was obtained in two types of experiments: serum titration and IC_50_ determination in the virus neutralization assay and by demonstration of serum IgG reactivity to SVPs of different HBV genotypes. In both tests, the VVX001 immunization was comparable to standard vaccination, yielding high anti-HBs levels (>2000 IU/L).

The protection against heterologous HBV genotypes is uneven and breakthrough symptomatic or occult HBV infections occur despite vaccination [48]. According to IgG and T cell reactivity to HBV preS1-peptides representing the NTCP attachment site, after VVX001 immunization, only the genotype G was notably less recognized, and this genotype was also not represented in the SVP experiment. However, infections with genotype G are rare, and chronic infections are only observed during coinfection with another HBV genotype [61]. Otherwise, the tested cross-reactivity was comparable for the common HBV genotypes. Of note, the preS-specific T cell and cytokine response measured in the study subject indicates that the response mainly consists of a CD4^+^ and to a low extent of CD8^+^ T cell response, accompanied by a mixed Th2/Th1 cytokine response. This response is compatible with the induction of a robust blocking antibody response and may be modified on demand by the use of different adjuvants or modes of vaccination if a more cytotoxic response is needed.

PreS-based HBV vaccines are of interest because they may be used as preventive and eventually also as therapeutic HBV vaccines. In fact, preS-specific antibodies may inhibit the infection of liver cells by preventing HBV from binding to its receptor NTCP. The preS1-derived lipopeptide bulevirtide (also known as Myrcludex B), which inhibits the NTCP–preS1 interaction, is now an approved drug [30]. Bulevirtide has the advantage of immediate action not requiring the functional immune response of the host, however, the disadvantages are the quick elimination time, interference with bile acid transportation in hepatocytes, and most importantly, lack of long-term protection if the treatment is discontinued. Therapeutic vaccination with preS-based vaccines such as VVX001 may have advantages over entry inhibitors blocking the preS1–NTCP interaction. Firstly, preS fusion constructs can be inexpensively obtained in gram amounts under good manufacturing practice conditions by recombinant expression in *E. coli* as soluble, pure, and stable proteins [62]. While the myristoylation of preS1-peptide is essential for the efficacy of bulevirtide, it does not seem to be important for the induction of HBV-neutralizing antibodies, as illustrated by the fact that BM32 produced in *E. coli* lacking myristoylation could induce neutralizing antibodies, as also shown by various monoclonal antibody studies [63].

Another advantage of preS-based vaccination is that active post-vaccination immunity is achieved by relatively short immunization schedules and could be subsequently boosted with just one or few immunizations. In this context, preS-based vaccination may also be considered to block co-infection with hepatitis D virus, a satellite viroid requiring HBV envelope proteins for replication. Hepatitis D is considered as the most severe chronic form of viral hepatitis due to quicker progression toward liver-related death and often requires liver transplantation [30]. Vaccination with preS induces polyclonal preS-specific antibodies that may prevent the formation of escape HBV mutants due to high conservation of the NTCP binding site between all common HBV genotypes A–J, as evidenced in our study and in our previous work [33]. Finally, another important application of preS-based vaccines like VVX001 might be the prevention of mother-to-child transmission (MTCT), which is responsible for a considerable rate of breakthrough infections in neonates, despite the application of both active and passive HBsAg-based immunization; the lack of the prognostically valuable markers and adequate follow-up of the neonates are further challenges demanding a strengthening of the existing measures to prevent MTCT [48,64]. Current guidelines include the standard HBV vaccination, antiviral treatment, and hepatitis B immunoglobulin (HBIG) administration [65]. However, considering the presence of circulating SVPs, both active vaccination targeting preS and the administration of preS-specific antibodies might be more successful in blocking breakthrough infections.

Regarding preventive vaccination, preS-based vaccines may especially be considered to overcome low- and non-responsiveness in subjects to HBsAg-based vaccines. In fact, between 5 and 20% of the persons currently vaccinated with HBsAg-based vaccines represent low- or non-responders [14,15,48]. It is a limitation of our study that only one person was tested, and more data are needed to test this approach. As a matter of fact, VVX001 is currently under evaluation in a clinical trial including, alongside healthy volunteers and HBV-infected individuals, a population of low- and non-responders to HBsAg-based vaccination (NCT03625934).

## 5. Conclusions

In conclusion, our study showed that vaccination with the preS-based vaccine VVX001 is safe and induced a robust HBV-neutralizing antibody response in a low-responder to HBsAg-based vaccines. VVX001 may therefore be considered as a promising candidate for HBV vaccination in low- or non-responders to currently available HBsAg-based vaccines. Further clinical studies will be required to test this hypothesis.

## Figures and Tables

**Table 1:** Quantification of the preS-specific IgG_1_ and IgG_4_ antibody concentrations.

Time Point	Date	IgG_1_, µg/mL	IgG_4_, µg/mL
		preS-specific	
Baseline (before VVX001 vaccination)	19 November 2018	6.19	0
1 month after the last injection	5 April 2019	1940.72	15
4 months after the last injection	26 June 2019	1347.55	16.4
8 months after the last injection	7 November 2019	868.05	4.4
12 months after the last injection	12 March 2020	679.51	0
		Peptide A-specific	
Baseline (before VVX001 vaccination)	19 November 2018	0	0
1 month after the last injection	5 April 2019	72.24	1.55
4 months after the last injection	26 June 2019	24.8	2.7
8 months after the last injection	7 November 2019	9.91	0
12 months after the last injection	12 March 2020	5.85	0
		Peptide B-specific	
Baseline (before VVX001 vaccination)	19 November 2018	0	0
1 month after the last injection	5 April 2019	23.42	0
4 months after the last injection	26 June 2019	9	0
8 months after the last injection	7 November 2019	4.45	0
12 months after the last injection	12 March 2020	2.85	0
		Peptide C-specific	
Baseline (before VVX001 vaccination)	19 November 2018	1.23	0
1 month after the last injection	5 April 2019	90.09	3.3
4 months after the last injection	26 June 2019	29.48	5
8 months after the last injection	7 November 2019	11.4	0
12 months after the last injection	12 March 2020	7.41	0

## Data Availability

All of the data supporting the findings of this study are available in the text, figures, tables, supplementary figures, or from the corresponding author upon reasonable request.

## Supplementary Materials

The following supporting information can be downloaded at: https://www.mdpi.com/article/10.3390/vaccines12101123/s1. Table S1: Features of the used HBV screening methods. Table S2: Lists of synthetic preS-derived peptides. Table S3: Anti-HBs antibody quantification and HBV screening parameters. Figure S1: Layout and exemplary measurements of the preS micro-array. Figure S2: Scheme of the in vitro virus neutralization assay. Figure S3: The preS-specific antibody response was dominated by IgG_1_. Figure S4: Phl p 5-specific IgG was lower than the preS-specific IgG levels. Figure S5: IgM and IgA levels specific for preS and preS-derived peptides. Figure S6: IgG_1_ and IgG_4_ reactivity to preS and preS-derived peptides. Figure S7: Additional outcome parameters of the in vitro HBV neutralization assays. Figure S8: PreS-specific cytokine responses.
