# Supplementary material for: The PreS-Based Recombinant Vaccine VVX001 Induces Hepatitis B Virus Neutralizing Antibodies in a Low-Responder to HBsAg-Based HBV Vaccines
Source: Vaccines (Basel). 2024 Sep 30;12(10):1123. doi: 10.3390/vaccines12101123 (PMC11511130; doi:10.3390/vaccines12101123)
Supplement: Supplementary file 1 [file vaccines-12-01123-s001.zip › vaccines-3146427-supplementary.pdf]

Table S1. Features of the used HBV screening methods.

Assay name	Test	Manufacturer	Reference values
HBs Ab-ELISA	Anti-HBs antibody (quantitative)	Dia.Pro Diagnostics (Sesto San Giovanni, Italy)	<20 mIU/mL: non/low-responder<br>20 - <100 mIU/mL: equivocal, booster vaccination recommended<br>≥ 100 mIU/mL: currently protected<br>(Vaccination guidelines in Austria: Impfplan Österreich 2023/2024; Version 2.0 from 14.05.2024 https://www.sozialministerium.at/Themen/Gesundheit/Impfen/Impfplan-%C3%96sterreich.html)
HBsAg-ELISA	HBs-antigen (qualitative)	Dia.Pro Diagnostics (Sesto San Giovanni, Italy)	Qualitative (positive/negative)
HBe Ag&Ab-ELISA	HBe-antigen (qualitative)	Dia.Pro Diagnostics (Sesto San Giovanni, Italy)	Qualitative (positive/negative)
HBc Ab-ELISA	Anti-HBc antibody (qualitative)	Dia.Pro Diagnostics (Sesto San Giovanni, Italy)	Qualitative (positive/negative)
Aptima® HBV Quant Assay	HBV-PCR (quantitative)	Hologic Inc., San Diego, CA, USA	Limit of detection:<br>< 10 IU/mL (negative)

Table S2. Lists of synthetic preS-derived peptides. Shown are the amino acid sequences of the peptides used in the assessment of preS-specific immune response.

PreS-derived peptides	Accession No.	Sequence
**Peptides mapping the N-terminal epitopes of preS**		
Peptide A (aa 1-29)	AAT28735	CMGGWSSKPRKGMGTNLSVPNPLGFFPDHQ
Peptide B (aa 30-61)		CLDPAFGANSNNPDWDFNPIKDHWPAANQVGVG
Peptide C (aa 17-51)		CSVPNPLGFFPDHQLDPAFGANSNNPDWDFNPIKDH
**Overlapping peptides spanning the whole preS sequence**		
P1 (aa 2-31)	AAT28735	GGWSSKPRKGMGTNLSVPNPLGFFPDHQLD
P2 (aa 22-51)		LGFFPDHQLDPAFGANSNNPDWDFNPIKDH
P3 (aa 42-71)		DWDFNPIKDHWPAANQVGVGAFGPGLTPPH
P4 (aa 62-91)		AFGPGLTPPHGGILGWSPQAQGILTTVSTI
P5 (aa 82-111)		QGILTTVSTIPPPASTNRQSGRQPTPISPP
P6 (aa 102-131)		GRQPTPISPPLRDSHPQAMQWNSTAFHQAL
P7 (aa 122-151)		WNSTAFHQALQDPRVRGLYFPAGGSSSGTV
P8 (aa 142-174)		PAGGSSSGTVNPAPNIASHISSISARTGDPVTN
**Peptides covering the NTCP attachment site of genotypes A–H**		
Genotype A	APD28359	GTNLSVPNPLGFFPDHQLDPAFGANSNNPDWDFNPIKDH
Genotype B	BAA88276	GTNLSVPNPLGFFPDHQLDPAFKANSENPDWDLNPHKDN
Genotype C	BAA32833	GTNLSVPNPLGFFPDHQLDPAFGANSNNPDWDFNPNKDH
Genotype D	BAD02320	GQNLSTSNPLGFFPDHQLDPAFRANTANPDWDFNPNKDT
Genotype E	BAC65105	GKNHSTTNPLGFFPDHQLDPAFRANTRNPDWDHNPNKDH
Genotype F	AAG49720	GQNLSVPNPLGFFPDHQLDPLFRANSSSPDWDFNKNKDN
Genotype G	BAB64320	GKNLSTSNPLGFLPDHQLDPAFRANTNNPDWDFNPKKDP
Genotype H	BAB69786	GQNLSVPNPLGFFPDHQLDPLFRANSSSPDWDFNTNKDN

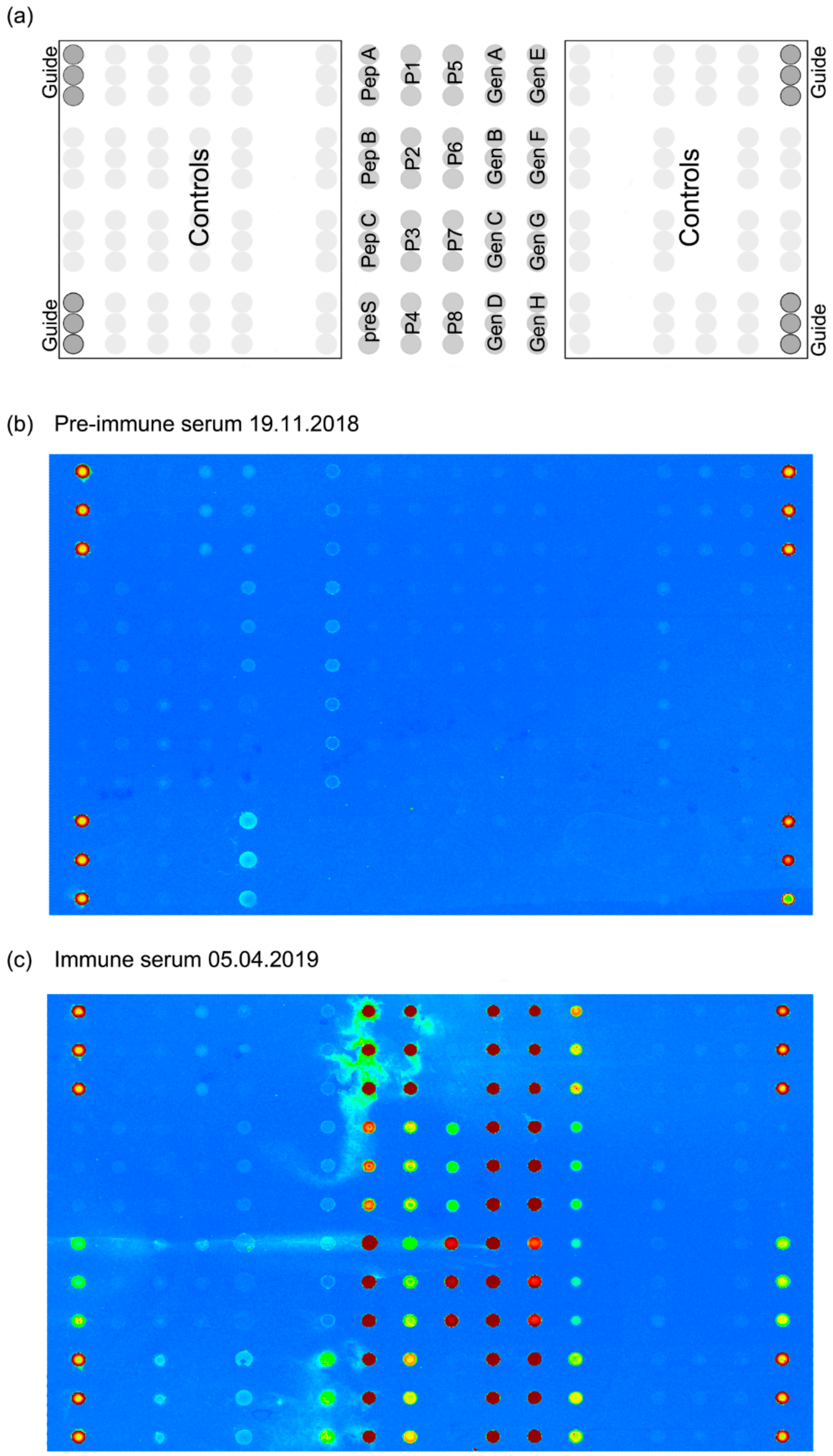

Figure S1. Layout and exemplary measurements of the preS micro-array. (**a**) Spotting scheme of a preS micro-array showing the order of dots (triplicates). (**b**,**c**) Scanned images depicting the fluorescence of the (b) pre-immune and (c) and post-vaccination serum sample.

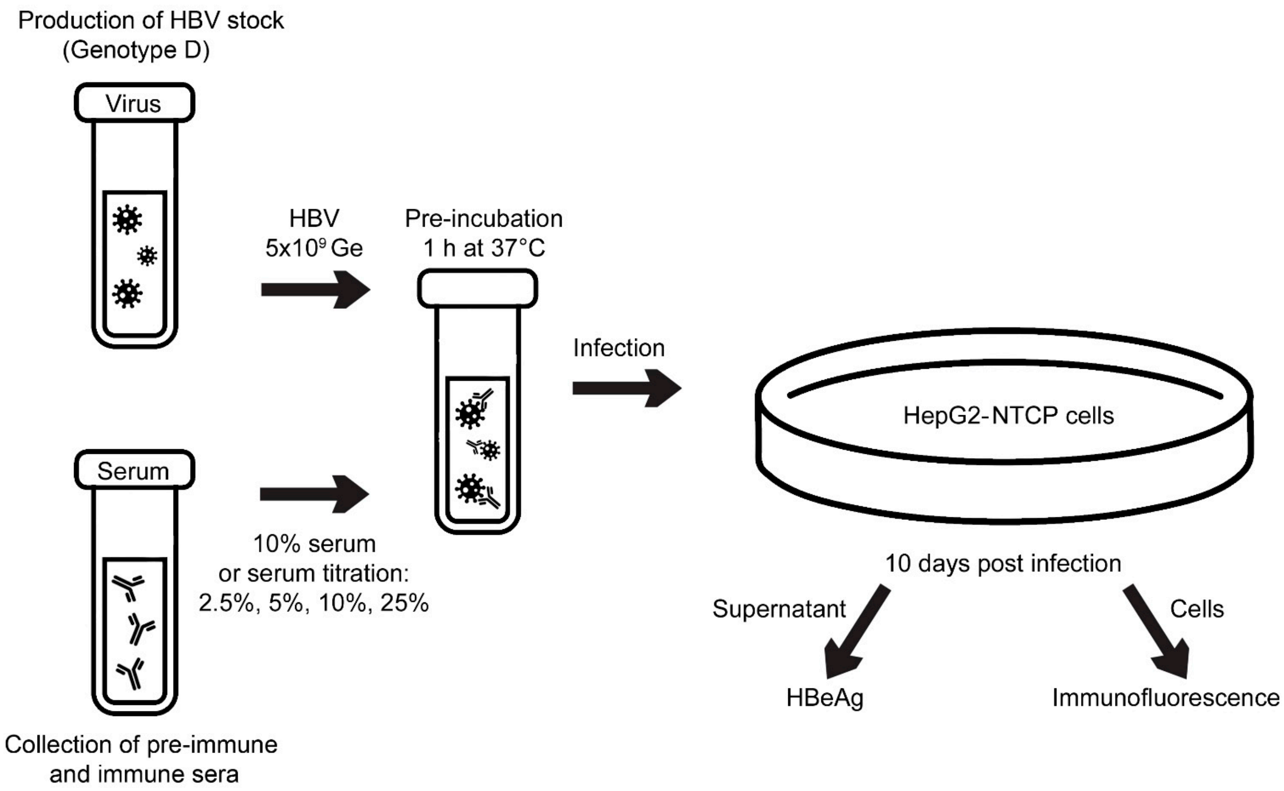

Figure S2. Scheme of the in vitro virus neutralization assay. A human hepatoma cell line (HepG2), inducibly expressing the HBV high affinity entry receptor NTCP, was infected with in vitro generated HBV (genotype D) after pre-incubation with immune sera at specified concentrations. HBV-infected cells secrete the soluble HBeAg, which is an accepted quantitative marker for productive HBV infection in cell culture experiments. To authenticate the generated HBV infectivity parameter HBeAg, the conventionally used qualitative immunofluorescence of HBV-infected cells was used. Here, newly produced intracellular HBV core protein (HBcAg) after HBV infection was stained and analyzed fully-automated to gain additional semi-quantitative data on the number of HBV-infected cells.

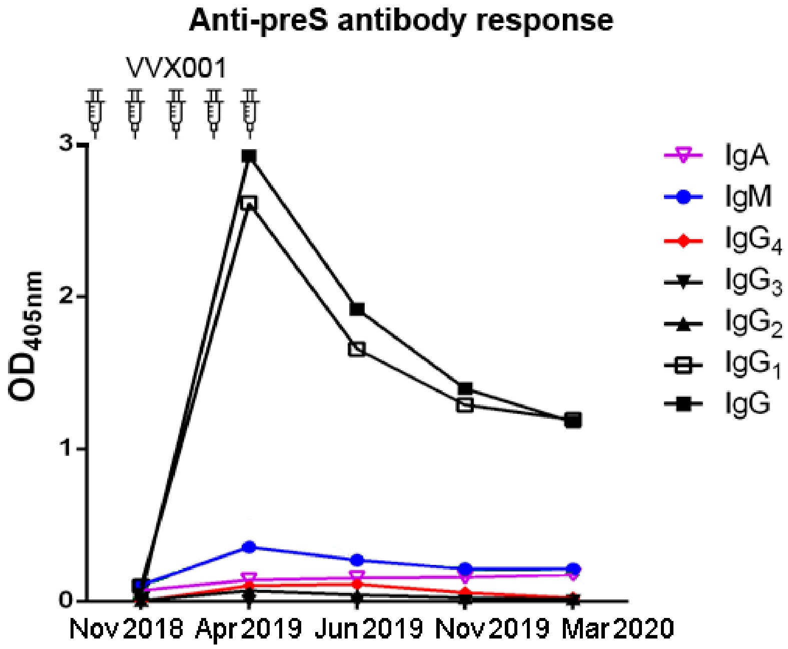

Figure S3. The preS-specific antibody response was dominated by IgG1. Shown are the OD values of preS-specific IgG, IgG1, IgG2, IgG3, IgG4, IgM, and IgA measured by ELISA (y-axis) over time.

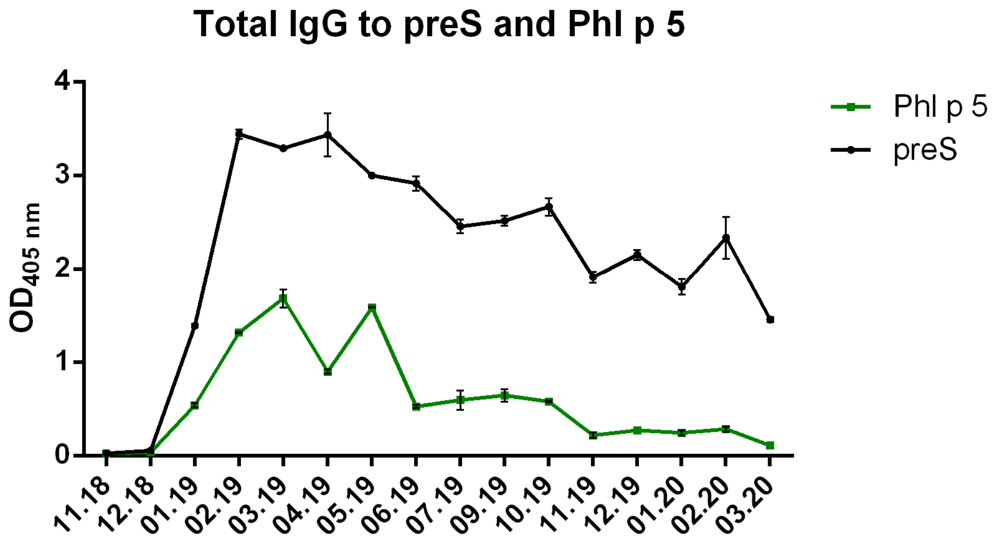

Figure S4. Phl p 5-specific IgG were lower than the preS-specific IgG levels. Shown are the OD values of the preS- and Phl p 5-specific IgG levels measured by ELISA over time.

Table S3. Anti-HBs antibody quantification and HBV screening parameters.

Serum Number	Date	Anti-HBs-Ab (mIU/mL)	HBs-Ag	HBe-Ag	Anti-HBc-Ab	HBV-PCR
1	08.02.2018	3	nd	nd	nd	nd
2	02.03.2018	88	nd	nd	nd	nd
3	06.04.2018	65	nd	nd	nd	nd
4	08.06.2018	75	negative	negative	negative	negative
5	09.08.2018	49	negative	negative	negative	negative
6	19.11.2018	40	negative	negative	negative	negative
7	14.12.2018	37	negative	negative	negative	negative
8	04.01.2019	45	negative	negative	negative	negative
9	11.01.2019	43	negative	negative	negative	negative
10	07.02.2019	32	negative	negative	negative	negative
11	08.03.2019	30	negative	negative	negative	negative
12	05.04.2019	27	negative	negative	negative	negative
13	29.05.2019	27	negative	negative	negative	negative
14	26.06.2019	19	negative	negative	negative	negative
15	26.07.2019	16	nd	nd	nd	nd
16	03.09.2019	13	nd	nd	nd	nd
17	01.10.2019	14	nd	nd	nd	nd
18	07.11.2019	11	nd	nd	nd	nd
19	04.12.2019	14	nd	nd	nd	nd
20	14.01.2020	13	nd	nd	nd	nd
21	11.02.2020	15	nd	nd	nd	nd
22	12.03.2020	12	nd	nd	nd	nd

mIU/mL: milli international units per milliliter. nd: not done.

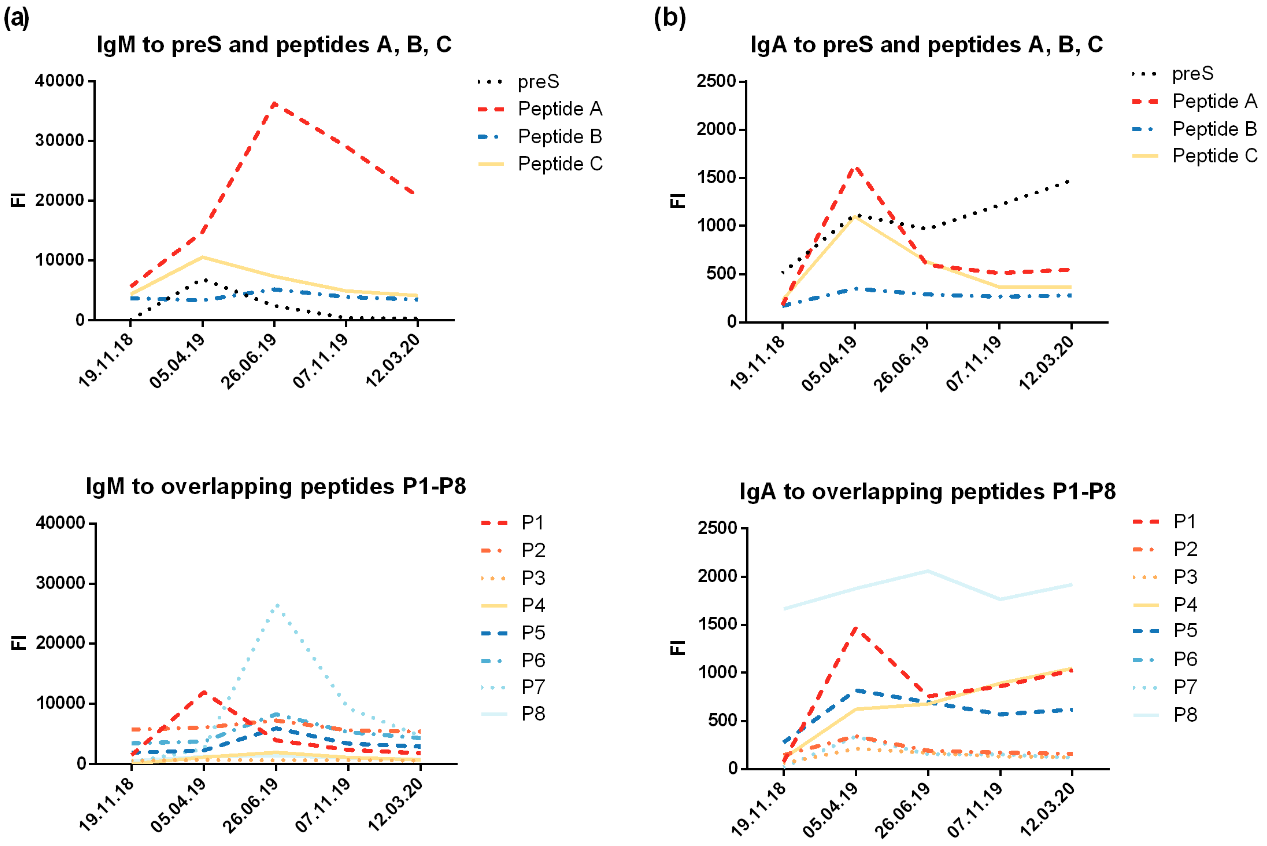

Figure S5. IgM and IgA levels specific for the preS and preS-derived peptides. Shown are the levels of preS/peptide-specific (**a**) IgM and (**b**) IgA (fluorescence intensity, FI) measured with the preS micro-array.

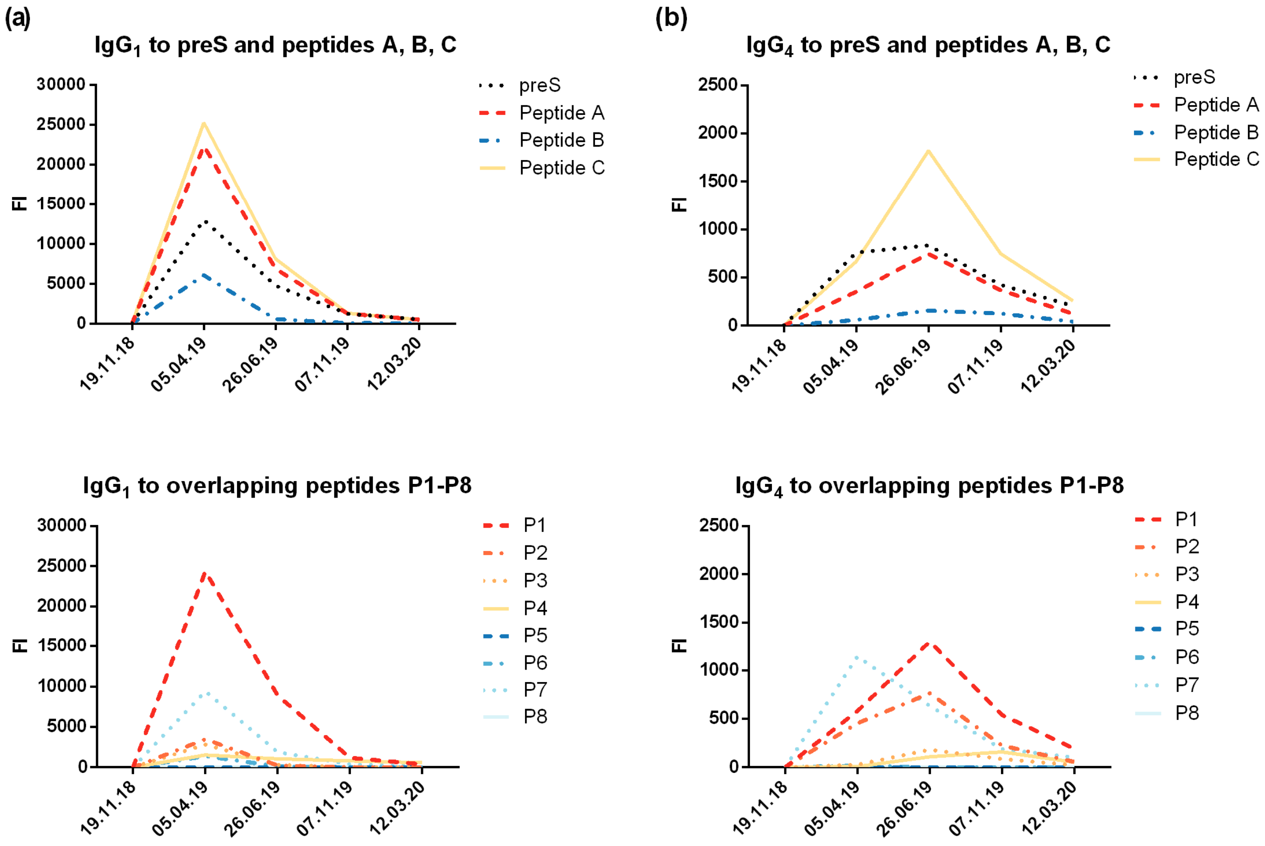

Figure S6. IgG1 and IgG4 reactivity to preS and preS-derived peptides. Shown are the levels of preS/peptide-specific (**a**) IgG1 and (**b**) IgG4 (fluorescence intensity, FI) measured with the preS micro-array.

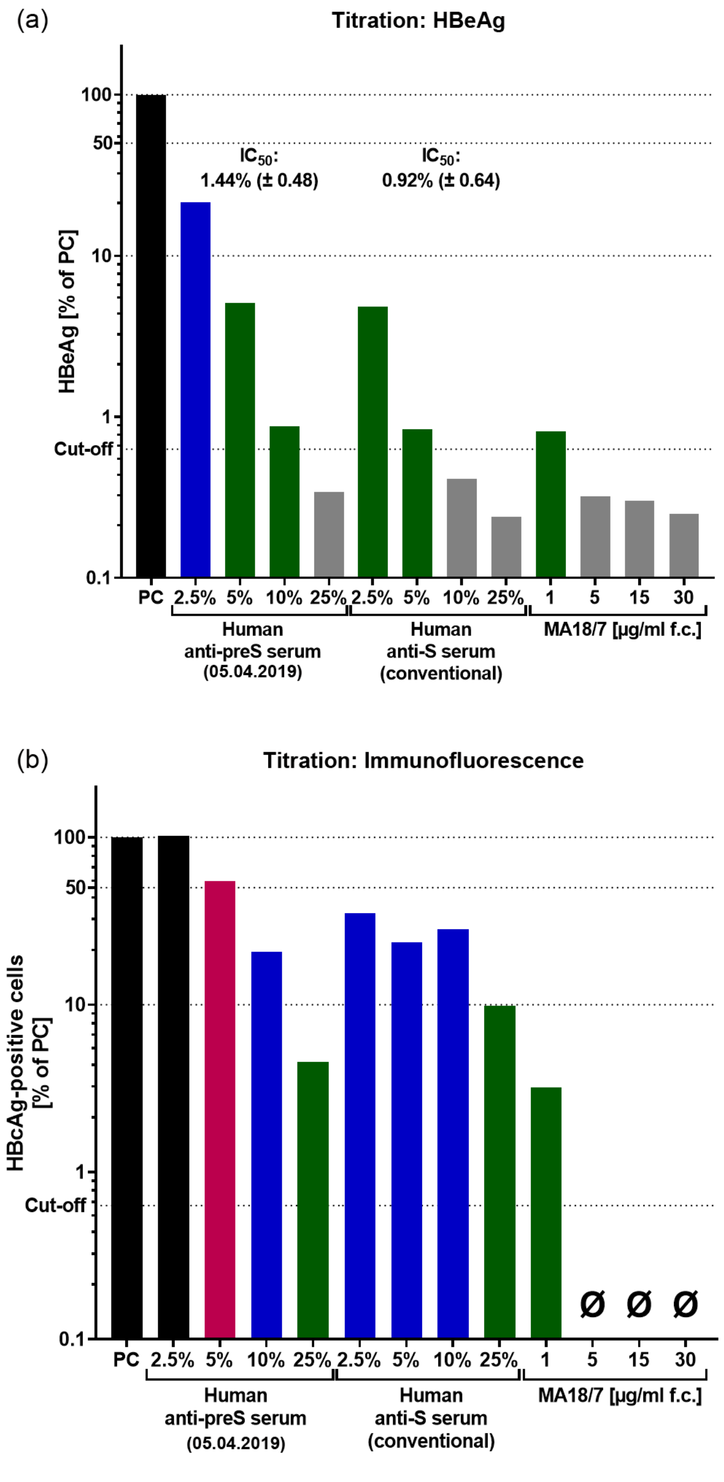

Figure S7. Additional outcome parameters of in vitro HBV neutralization assays. (b) HBeAg secretion and (**b**) de novo expressed HBcAg (baseline correction of immunofluorescence was performed by subtracting the value of the uninfected control) of infected cells after the pre-incubation of HBV inoculum with titrated sera after vaccination. Data are presented as % compared to the positive control (PC, infection w/o serum pre-incubation). Neutralization: ≥90% (strong neutralization; green), ≥50% (partial neutralization; blue), ≥10% (weak neutralization; magenta); HBeAg below cut-off (grey). Ø = 0% HBcAg-positive cells after baseline-correction.

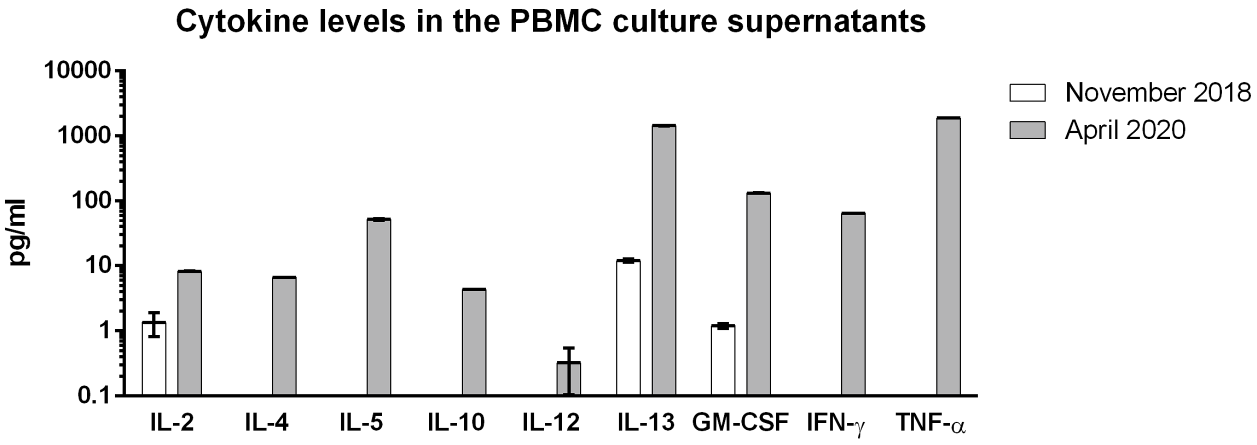

Figure S8. PreS-specific cytokine responses. Shown are the levels of IL-2, IFN-γ, IL-4, IL-5, IL-10, IL-13, GM-CSF, and TNF-α measured in the supernatants of seven-day PBMC cultures stimulated with preS at the baseline (November 2018) and one year after the last injection (April 2020) after subtraction of the medium control.
